# Planar Interdigitated Aptasensor for Flow-Through Detection of *Listeria* spp. in Hydroponic Lettuce Growth Media

**DOI:** 10.3390/s20205773

**Published:** 2020-10-12

**Authors:** Raminderdeep K. Sidhu, Nicholas D. Cavallaro, Cicero C. Pola, Michelle D. Danyluk, Eric S. McLamore, Carmen L. Gomes

**Affiliations:** 1 Department of Biological & Agricultural Engineering, Texas A&M University, College Station, TX 77843, USA; aligarh01@gmail.com; 2 Agricultural & Biological Engineering, Institute of Food and Agricultural Sciences, University of Florida, Gainesville, FL 32611, USA; ncavallaro@ufl.edu; 3 Department of Mechanical Engineering, Iowa State University, Ames, IA 50011, USA; cicerocp@iastate.edu; 4 Food Science and Human Nutrition, Institute of Food and Agricultural Sciences, University of Florida, Gainesville, FL 32611, USA; mddanyluk@ufl.edu

**Keywords:** food safety, electrochemical sensing, foodborne pathogen, fresh produce, interdigitated electrodes, sensor analytic point solution, SNAPS

## Abstract

Irrigation water is a primary source of fresh produce contamination by bacteria during the preharvest, particularly in hydroponic systems where the control of pests and pathogens is a major challenge. In this work, we demonstrate the development of a *Listeria* biosensor using platinum interdigitated microelectrodes (Pt-IME). The sensor is incorporated into a particle/sediment trap for the real-time analysis of irrigation water in a hydroponic lettuce system. We demonstrate the application of this system using a smartphone-based potentiostat for rapid on-site analysis of water quality. A detailed characterization of the electrochemical behavior was conducted in the presence/absence of DNA and *Listeria* spp., which was followed by calibration in various solutions with and without flow. In flow conditions (100 mL samples), the aptasensor had a sensitivity of 3.37 ± 0.21 kΩ log-CFU^−1^ mL, and the LOD was 48 ± 12 CFU mL^−1^ with a linear range of 10^2^ to 10^4^ CFU mL^−1^. In stagnant solution with no flow, the aptasensor performance was significantly improved in buffer, vegetable broth, and hydroponic media. Sensor hysteresis ranged from 2 to 16% after rinsing in a strong basic solution (direct reuse) and was insignificant after removing the aptamer via washing in Piranha solution (reuse after adsorption with fresh aptamer). This is the first demonstration of an aptasensor used to monitor microbial water quality for hydroponic lettuce in real time using a smartphone-based acquisition system for volumes that conform with the regulatory standards. The aptasensor demonstrated a recovery of 90% and may be reused a limited number of times with minor washing steps.

## 1. Introduction

The Centers for Disease Control and Prevention (CDC) estimates that up to 48 million illnesses, 128,000 hospitalizations, and 3000 deaths in the United States are caused by foodborne pathogens each year [1]. Despite strict regulations to control the presence of foodborne pathogens in the food supply, the incidence of illnesses and deaths from food by pathogens results in an estimated cost of $14.6 to $16.3 billion per year [2]. These estimates do not include the economic burden of food waste that is linked to microbial contamination, which is a serious problem in the United States [3].

The contamination of fresh produce (e.g., leafy greens) by bacteria is a major problem [4,5], and water is one of the main route of infections for human exposure [6,7]. The food safety modernization act recently enacted the final compliance deadline for the produce safety rule (PSR) designed to address some issues related to this problem [8,9,10]. The PSR is focused on generic *Escherichia coli* as an indicator organism, but current efforts in many research labs are underway to also consider the direct measurement of pathogenic microorganism. Among these, *Listeria* spp. is one major concern related to food recalls.

*Listeria* spp. are Gram-positive bacteria that are ubiquitous in soil [11], and they are also found in hydroponic systems [12], processing environments [13], and animal operations [14]. *Listeria* are found across the supply chain (i.e., from “farm to fork”); thus, tracking this foodborne pathogen is challenging [15]. For instance, *Listeria monocytogenes* is the bacteria responsible for listeriosis, the third leading cause of death from food poisoning [11,12]. Given the transient nature of *Listeria* in the food chain, a variety of sensors and biosensors have been developed in the last few decades to monitor pathogens associated with food safety [16,17,18,19,20,21,22,23].

The contamination of lettuce by bacteria such as *L. monocytogenes* and fecal coliforms is a major issue, including the contamination of hydroponic systems where this pathogen is known to attach to leaves at a higher rate than in soil-based culture [24,25,26]. Lettuce is the most valuable leafy crop in the U.S. [27]; thus, contamination is a major concern in multiple aspects of the supply chain. Sensors are one important tool used to assess microbiological safety in the supply chain, and they have applications in irrigation water quality monitoring as well as direct analysis of food samples.

Among *Listeria* biosensors, one of the most promising biosensors, also known as aptasensors, utilizes a DNA aptamer (47-mer) that targets a cell-surface invasion protein found on *Listeria* spp. (Internalin A, InlA). Ohk et al. [21] tested this InlA 47-mer using a fiber optic sensor and showed comparable performance to antibodies targeting *Listeria* spp. Hills et al. [28] also used the 47-mer discovered by Ohk et al. [21] to detect *Listeria* with an electrochemical sensor based on nanoplatinum–graphene electrodes.

In this work, we demonstrate the development of a *Listeria* biosensor using platinum interdigitated microelectrodes (Pt-IME) biofunctionalized with *Listeria*-specific aptamer (47-mer) and incorporate the sensor into a particle/sediment trap for real-time analysis of irrigation water in hydroponic media. Furthermore, we demonstrate this sensing device using a smartphone-based signal acquisition system [29] for rapid on-site analysis of water quality in hydroponics with a response time of only 27 min. Pt-IME with different finger spacing of 25, 50, and 100 μm were fabricated and tested to select the optimum finger spacing for improved performance during electrochemical sensing (i.e., high signal-to-noise ratios, fast response times, and enhanced reaction–diffusion kinetics). The Pt-IME were biofunctionalized with a *Listeria*-specific aptamer through thiol–metal bonding at optimum loading concentration, followed by calibration in various media in stagnant and high-flow conditions. In addition, sensor hysteresis was investigated for direct reuse (washing with strong basic solution) and regeneration (using Piranha solution followed by aptamer biofunctionalization). The resulting biosensor is capable of sensing *Listeria* spp. in buffer solution and real food (vegetable broth) in stagnant media, as well as in a high flow-through system of irrigation water in hydroponic systems at relevant concentrations to regulatory standards for assessing agricultural water quality. Additionally, this biosensor has a high level of recovery and can be reused a number of times with minor washing steps.

## 2. Experimental

### 2.1. Materials, Reagents and Equipment

Silicon wafer (4 inches) with a wet thermal oxide thickness of 300 nm and a resistivity of 0.001–0.005 Ω cm was purchased from University Wafer (Fremont, CA, USA). Platinum pellets, Pt, 99.99% pure, 1/8” diameter were obtained from Kurt J. Lesker (Jefferson Hills, PA, USA). A non-UV sensitive polymer (LOR 3A) was purchased from MicroChem (Newton, MA, USA). AZ 5214 E-positive photoresist, AZ 726 MIF-standard photoresist developer, and AZ 400T-photoresist stripper were purchased from EMD Performance Materials (Sommerville, NJ, USA). Mylar masks were purchased from CAD/Art Services, Inc (Bandon, OR, USA). Silver conductive epoxy was purchased from Allied Electronics (Austin, TX, USA). Silver/silver chloride (Ag/AgCl) standard reference electrode and platinum auxiliary electrodes were purchased from BASi (West Lafayette, IN, USA). Details of the materials may be found in the (Supplemental section Table S1).

Hydrogen peroxide 3% (wt), sulfuric acid (H_2_SO_4_), potassium nitrate (KNO_3_), potassium chloride (KCl), potassium ferrocyanide trihydrate (K_3_Fe(CN)_6_), and phosphate buffer saline (PBS) were purchased from Sigma Aldrich (St. Louis, MO, USA). Buffered peptone water (BPW) was purchased from HiMedia (Mumbai, India). *Listeria innocua* (ATCC 33090) was purchased from American Type Culture Collection (Manassas, VA, USA) and cultured in tryptose phosphate broth (TPB) bought from HiMedia (Mumbai, India). Oxford *Listeria*-selective agar and Oxford *Listeria*-selective supplement were purchased from EMD Performance Materials (Sommerville, NJ, USA). Petrifilms were purchased from 3M (aerobic plate count, St. Paul, MN, USA). Vegetable broth (Swanson, Campbell Soup Company, Camden, NJ, USA) was purchased in a local grocery store.

The equipment used for the fabrication of Pt-IME included the following: Verteq photoresist spinner, Karl Suss MA6 mask aligner, Lesker PVD 75 e-beam evaporator, and Aggiefab dicing saw. All clean room work was conducted at the Aggiefab facility at Texas A&M University (College Station, TX, USA). A CHI 600E potentiostat (Austin, TX, USA) with CHI6044e software or handheld potentiostat (ABE-STAT [29]) with Samsung Galaxy tablet was used for electrochemical analysis as noted. A Bruker Dektak Profilometer (Tucson, AZ, USA) was used to quantify electrode features.

### 2.2. Bacteria Strains and Culture

*L. innocua* ATCC 33090 was used as a non-pathogenic surrogate for *L. monocytogenes*, since they are found in analogous environments and present similarities when growing in leafy greens [30]. *L. monocytogenes* ATCC 15313 was used for the selectivity test in complex media. *Listeria spp.* cultures originally stored at −80 °C were revived twice in TPB for 24 h at 37 °C. After activation, bacteria cultures were kept in the refrigerator (5 °C), and weekly transfers were made in TPB followed by incubation at 37 °C for 24 h until use. Before sensing experiments, serial dilutions were made in BPW to achieve 10–10^6^ CFU mL^−1^, and plate counting on Oxford agar was used to confirm the bacterial concentration following protocol described by USFDA [31].

### 2.3. Electrochemical Characterization

Electroactive surface area (ESA), heterogenous electron transfer (HET) constant, current density, and impedimetric parameters were analyzed before and after aptamer addition. Cyclic voltammetry (CV) was used to determine ESA and HET constant based on our previous work [32]. CV was performed in 4 mM KFe(CN)_6_ with 1 M KNO_3_ at a switching potential of 0.75 V versus a Ag/AgCl reference electrode. DC potential amperometry (DCPA) was used to determine current density toward H_2_O_2_. Impedimetric parameters (charge transfer resistance, diffusive resistance, solutions resistance, and capacitance) were determined by electrochemical impedance spectroscopy (EIS). All tests were performed using 4 mM K_4_Fe(CN)_6_ with 1 M KCl. A DC potential bias of 200 mV was applied across the frequency range from 1 Hz to 100 kHz and an AC amplitude of 100 mV for EIS. For baseline characterization, Nyquist, Bode, and Phase diagrams were developed and analyzed using Zman software based on previously published techniques [28,32,33,34,35,36].

### 2.4. Pt-IME Fabrication Procedure

The Pt-IME sensor design was based on an array of comb fingers connected to larger contact pads. Electrode arrays with different geometric between-fingers gaps were designed to produce electrode spacing (S) dimensions of 25, 50, and 100 μm (Supplementary Figure S1). Pt-IME were fabricated using a one-mask fabrication process consisting of photolithography, dual layer lift-off, and electrodeposition. One wafer consisted of eight devices in total with two replicas. Each Pt-IME array had a width of 25 μm and height of 115 µm with a total active area of 0.81 cm^2^ and bonding pads 200 × 200 μm. Within the total active area of 0.81 cm^2^, the number of electrodes changed for each IMEs with different electrode gaps. The thickness of wet thermal oxide was 90 nm. Pt-electrode thickness was designed to be 110 nm based on the optimum height for ferrocyanide redox reactions as originally determined by Min and Baeumner [37].

Mylar masks were used to delineate interdigitated microelectrodes array and bonding pads; details can be found in the supplemental section on mask design and feature size. Silver conductive ink was used for wire bonding and allowed to dry for at least 24 h prior to analytical testing. See Supplementary Table S1 for additional details.

### 2.5. In Silico Model of Pt-IME Capacitance

An in silico model (COMSOL Multiphysics, Burlington, MA) of Pt-IME capacitance was developed by combining the model developed by Jun et al. [38] with the model by Oberländer et al. [39] using Equation (1). The model estimated the electric field and used the calculated capacitance (C) to compare with impedance data for various IME geometries.

(1) $$C=L\left(N-1\right)*\left[\left(\frac{\varepsilon_{0}\varepsilon_{r}}{2}\right)*\left(\frac{K*{\left(1-k^{2}\right)}^{0.5}}{K\left(k\right)}\right)+\left(2\varepsilon_{0}\varepsilon_{r}\right)*\left(\frac{w}{s}\right)\right]$$

where N = number of electrodes, *ε*_0_ = permittivity of vacuum 8.851 × 10^−12^ As Vm^−1^, *ε_r_* is the total relative permittivity surrounding the electrodes, *K*(*k*) = impact of fringing field, *k* = periodic structure of electrode geometry as defined by Oberländer et al. [39], and the geometrical parameters are L, *w*, and *S* (Supplementary Figure S1).

### 2.6. Biofunctionalization of Pt-IMEs with Aptamers

Prior to aptamer adsorption, IMEs were cleaned with Piranha solution (3:1 sulfuric acid: hydrogen peroxide) for one minute, washed with deionized (DI) water, and then air dried. Pt-IME were biofunctionalized with thiol-tagged DNA 47-mer that targets a cell surface protein (InIA) on *L. monocytogenes* [21]. A thiol tag and C6 spacer ((CH_2_)_6_OH) were inserted at the 3*′* end for direct adsorption to platinum electrodes vial metal–thiol bonding [28]. GeneLink (Hawthrone, NY) supplied custom oligonucleotides in desalted, lyophilized, and disulfide protected form. Dithiothreitol (DTT) was used to reduce the SH group (i.e., deprotect) for adsorption based on the manufacturer’s recommendation (GeneLink) [40]. Briefly, 100 mM DTT solution in sodium phosphate buffer (pH = 8.5) for 1 h at room temperature was mixed with the protected aptamers. Trace DTT residue was removed by the addition of sodium acetate per the protocol, which was followed by ethanol precipitation to isolate the thiolated aptamer. Briefly, 1.5 mL of absolute ethanol was added; then, the suspension was vortexed and placed for 20 min in a freezer at −80 °C. Next, the suspension was centrifuged for 10 min at 10,800× *g*. After removal of the supernatant, the deprotected aptamer was dried under vacuum (101.6 kPa) at room temperature for 20 min. Aptamers were re-suspended in 10 mM Tris, 1 mM ethylenediaminetetraacetic acid (EDTA), pH 7.5 buffer (TE buffer). Aptamer stock solutions (100 μM) were diluted as needed, and 65 μL was drop cast to biofunctionalize Pt-IMEs (two-hour binding time).

For determining aptamer loading, EIS and CV were conducted in a solution of PBS (pH 7.4) using a two-electrode setup with an AC potential of 100 mV at a frequency range of 1 Hz to 100 kHz. All CV studies were performed in 4 mM KFe(CN)_6_ with 1 M KNO_3_ in distilled water. EIS and CV plots were analyzed based on established protocols [28,41].

### 2.7. Protocol for Bacteria Detection

Pt-IME biosensors were immersed in test solution as noted, and three initial EIS sweeps were conducted to stabilize the dielectric layer and sensor signal. Tests were initiated when the baseline impedance changed by less than 1%. Once stabilized, an aliquot of BPW with cultured cells solution was added to achieve bacteria concentration ranging from 10 to 10^6^ CFU mL^−1^. After adding bacteria, the sample was stirred for 1 min; impedance measurements were taken after the stirring was turned off and the suspension was stagnant. The capacitor stabilizer was initiated, and the electrodes were grounded to minimize charge buildup onto the Pt-IMEs.

Nyquist plots were analyzed using a spreadsheet model coupled to ABE-STAT using Equation (2) based on a Randles equivalent circuit with Chi^2^ fitting. For validation of the custom equivalent circuit model, Zman software was used according to Hills et al. [28] and Burrs et al. [33].

(2) $$Z=R_{s}+\frac{1}{i\omega C_{dl}+\frac{1}{R_{ct}+\frac{R_{w}}{\sqrt{\omega }}-i\frac{R_{w}}{\sqrt{\omega }}}}$$

where *Z* = impedance, *R_s_* = solution resistance, *ω* = angular frequency, *C_dl_* = capacitive double layer, *R_ct_* = charge transfer resistance, *R_w_* = Warburg resistance, and *i* = current.

EIS plots were used to analyze biosensor response based on the methods by Hills et al. [28]. In summary, Bode plots were used to assess change in impedance, and Nyquist plots were used to assess changes in charge transfer resistance. Other electrical parameters (*R_s_*, *ω*, *C_dl_*, and *R_w_*) were not significant drivers of the output signal based on linear regression analysis. Sensitivity was calculated as the linear slope of calibration plots prepared using Bode plots at a cutoff frequency of 1 Hz. Selectivity was determined by analyzing sensitivity toward *L. innocua* in the range of 10 to 10^6^ CFU mL^−1^ in the presence of non-*Listeria* targets, as noted. After analysis, the biosensor was washed and the impedance was re-analyzed.

### 2.8. Hysteresis Testing

Pt-IMEs were cleaned with Piranha solution (3:1 sulfuric acid:hydrogen peroxide) under a chemical hood. The biosensor was carefully immersed in the solution for one minute, taking care not to expose the bonding pads to the Piranha solution. Next, the biosensor was thoroughly washed with DI water for one minute to ensure the proper removal of Piranha solution residues from the surface and then air dried. Cleaned Pt-IME were coated with aptamers and tested for potential reuse and for hysteresis analysis.

### 2.9. Analysis of Hydroponic Water

A RainForest modular 318 aeroponic system with Vortex sprayer was used to grow lettuce based on Marhaenanto et al. [42]. The main reservoir of the hydroponic system was 65 L, and the conical vortex sprayer was operated at 1200 rpm. Hydroponic lettuce (*Lactuca saliva*) was cultivated using 7.6 cm diameter plastic seed cups with CocoTek liners and expanded clay pellets (Mr. Stacky Hydroponic Center, Lake City, FL, USA). A photoperiod of 8 h was adopted, where lighting was based on full spectrum light-emitting diode (LED) grow lights (75 W equivalent). Nutrient solution (Liquid Plant Food Big Bloom, Fox Farm Organic Gardening, Arcata, CA, USA) was replaced every 7 days based on manufacturer’s recommendations. Growth media was sterilized according to our previous methods [43].

A particle trap was spliced into a 3/4” OD Tygon tube and attached to a submersible pump for Pt-IME measurements in the hydroponic system. The particle trap had a stainless-steel mesh (#50; 300 μm mesh) within the inner chamber, and the Pt-IME was fixed within this mesh strain for direct contact with the water prior to filtration in the particle trap. The trap was customized for Pt-IME analysis by drilling two small holes on the top of the plastic housing and threading male–male Dupont Wire (Arduino) through the hole. The holes in the plastic body were sealed with rubber sealant (FlexSeal, Weston, FL, USA), and the inner pins were soldered to the Pt-IME bonding pads. The lead wires were insulated with nail polish and dried overnight, and then the trap was fixed to the housing and sealed via the threaded fitting (Supplementary Figure S2).

For simulating contamination, 10 mL of *L. innocua* suspension was injected into a T-junction placed upstream of the particle trap/Pt-IME apparatus. The sump pump in the reservoir pumped continuously for 5 min after the injection of *L. innocua*. After 5 min of continuous flow, the pump was turned off for EIS analysis with a custom handheld potentiostat [29,44] and data were collected from the Pt-IME biosensor in the particle trap.

After each analysis, the Pt-IME was washed by flushing the particle trap with sterile growth media at a flowrate of 5 mL min^−1^ for 10 min. After washing, a subsequent aliquot of *L. innocua* was injected, and the analysis was repeated. All measurements were recorded at room temperature (24.5 ± 0.6 °C).

### 2.10. Statistical Analysis and Portfolio Analysis

JMP Software v. 11 (SAS Institute, Cary, NC, USA) was used for all statistical analyses. Means, error bars, and standard deviations were calculated based on triplicate tests. Differences between variables were tested for significance using one-way analysis of variance (ANOVA) and significantly different means (*p* < 0.05) were separated using a Tukey honest significant difference (HSD) test. Impedance measurements were used to determine the limit of detection (LOD), sensitivity, and sensing range. Limit of detection was calculated using the 3σ method based on the signal to noise (S/N) ratio [32,45]. Sensitivity was determined by the slope of the calibration plots as described previously. Sensing range was determined based on the linear increase of signal over a range of bacteria concentration [46]. Change in impedance was calculated from the biosensor’s baseline (no bacteria). Finally, detection time was determined as the time of bacteria incubation on the biosensor (15 min) and the EIS measurement time (2 min).

To compare the performance of various sensor geometries, a multicriteria decision analysis (MCDA) was conducted using the open source analytical hierarchy process by Goepel [47] modified by swing weights as reviewed by Lai et al. [48] The pairwise comparison was based on a threshold for acceptance of inconsistency (α) of 0.1.

## 3. Results and Discussion

**Figure 1:**
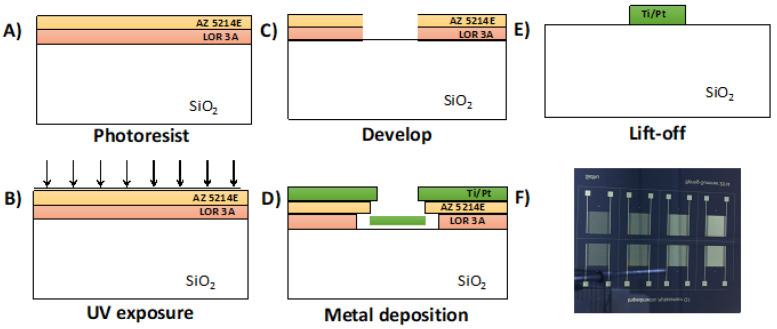
Microfabrication process for platinum interdigitated microelectrodes (Pt-IME) on SiO_2_ wafers. (**A**) Photoresist deposition, (**B**) UV exposure with IME mask, (**C**) Development of resist, (**D**) Ti/Pt deposition by chemical vapor deposition (CVD), and (**E**) Pattern lift off. (**F**) Photographs of Pt-IME array with eight electrodes.

The microfabrication process of the Pt-IME with different gap sizes on silicon is presented in Figure 1. Profilometer measurements indicated that the gap spacing was slightly smaller than design dimensions, and the error increased for smaller electrode gap sizes (Supplementary Figure S3 and Table S2). Moreover, previous studies [37,49,50] showed that the number of fingers in IME has no significant effect on the S/N ratio, and the signal value is proportional to the total IME surface area (A_s_), thus the choice to base the comparative study on equivalent A_s_.

### 3.1. In Silico Pt-IME Model

A basic dielectric model was developed for estimating the Pt-IME capacitance for various electrode configurations using a simplified approach based on Jun et al. [38] and Oberländer et al. [39]. The in silico model predicted that the electric field decreases with increasing gap size for the geometries tested here, and the minimum number of fingers was approximately 20 for all geometries (Supplementary Figure S4). According to the simple model, the optimum gap spacing is 25 μm, which also produces the maximum cell constant without constricting the dielectric field (Supplementary Figure S5), although the model did not consider electrode height or the complexity of the solution to keep the computational requirements reasonable. A simulation of the electrical field at the surface of the Pt-IME conformed to expected behavior (the power law exponent was 0.39), which is close to the 1/3 power law predicted by more advanced in silico models that show the intensity of an electric dipole field falls off with the cube of distance. Potential sources of error are the electrode height and the electrolyte complexity. Bäcker et al. [51] compared planar and 3D IME for sensing and expanded on this basic model by considering a dielectric barrier between the electrode fingers. This approach offers potential improvement of the dielectric field if the geometry can support controlled immobilization of aptamer for promoting cell capture. In this study, only planar electrodes were used to provide the baseline evidence for the detection of bacteria in complex samples such as hydroponic media under flowing conditions. Detailed electrochemical characterization was conducted to confirm the results of the in silico model regarding optimum gap spacing.

### 3.2. Electrochemical Characterization

**Figure 2:**
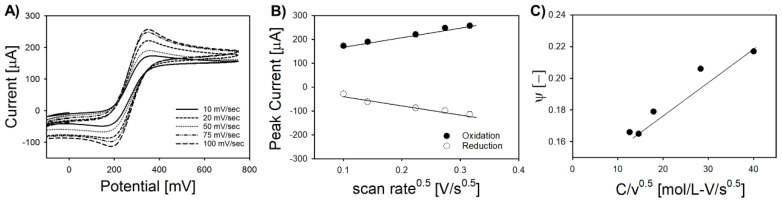
Electrochemical characterization of Pt-IME for gap spacing of 50 μm. (**A**) Representative cyclic voltammograms in 4 mM K_4_Fe(CN)_6_ + 1 M KNO_3_ at room temperature. (**B**) Randles–Sevcik plots for oxidative and reductive peak current indicate diffusion-limited transport to the Pt-IME. (**C**) Nicholson plots for the determination of heterogenous electron transfer (HET) constant (k^0^). Full analysis of all Pt-IME electrode gap spacing (with replicates) can be found in the supplemental section.

The electrochemical behavior of Pt-IME with various gap geometry was analyzed by determining ESA, HET constant, current density, amperometric response, and impedimetric response. Figure 2 shows representative CV plots (Figure 2A), Randles–Sevcik plots (Figure 2B), and Nicholson plots (Figure 2C) for Pt-IME with 50 μm gap spacing (see Supplementary Figure S6 for all data including other gap spacing). The ESA for IME with a 50 μm gap spacing (0.14 ± 0.02 cm^2^) was four times higher than IME with a gap spacing of 25 μm (0.04 ± 0.01 cm^2^) and significantly higher than IME with a gap spacing of 100 μm (0.11 ± 0.02 cm^2^). The HET constants (k^0^) for Pt-IME with 50 μm (34.6 ± 9.1 cm s^−1^) and 100 μm (44.2 ± 10.2 cm s^−1^) were significantly higher than 25 µm gap spacing (7.9 ± 6.4 cm s^−1^) but not significantly different compared to one another (*p* > 0.05). In this baseline characterization, a relatively high concentration of supporting electrolyte (1 M KNO_3_) was used to reduce interfacial electron transfer barrier and improve redox kinetics. Preliminary studies show that low electrolyte concentration led to relatively low Faradaic current, shifting the redox peaks toward the cathodic region and reducing the differential potential (ΔE), and thus HET constant, to values that were not significantly different among electrodes of varying gap size (*p* > 0.05).

DCPA plots for determining current density are shown in Supplementary Figure S7. For a gap spacing of 25 μm, charge overflow occurred in DCPA preliminary experiments when a DC polarization potential of +500 mV was applied. Thus, data were acquired at a DC potential of +280 mV to avoid cross-talk. Reducing gap spacing is desirable for bacteria detection, as large gap spacing can leave dead zones where bacteria do not interact with the recognition agent. However, reducing gap size must be a careful consideration as there is a well-established relationship between gap spacing and cross-talk in IME [52,53,54,55]. The average current density for Pt-IME with a gap spacing of 50 μm (149 ± 20 μA mM^−1^ cm^−2^) was significantly higher than the gap spacing of 25 μm (58 ± 19 μA mM^−1^ cm^−2^) and 100 μm (75 ± 12 μA mM^−1^ cm^−2^), which corroborates with ESA results. The response time for all Pt-IME was 5 s for all experiments. The average ESA, HET, and current density (toward H_2_O_2_) are shown in Supplementary Table S3.

EIS was used to determine the baseline electrochemical characteristics for each Pt-IME gap spacing using low amplitude sinusoidal modulation with a sweeping frequency of 1 Hz to 100 kHz. Based on the peak potential observed in CV (400 mV), a bias potential equal to *E_p_*/2 (200 mV) was applied for all impedance tests. Nyquist plots and Bode plots confirmed that charge overflow for 25 μm gap spacing was significant, leading to significant cross-talk. Pt-IME with a 50 μm gap spacing had a solution resistance of 95 ± 12 Ω, charge transfer resistance of 39 ± 7 kΩ, Warburg resistance of 5 ± 1 Ω s^−0.5^, and capacitive double layer of 8 ± 2 pF (all parameters were estimated using a Randles equivalent circuit). Pt-IME with 50 μm gap spacing had an impedance of 0.5 kΩ at 1 Hz cutoff frequency, which was 25% lower than the impedance for 100 μm gap spacing. Charge transfer resistance for 50 μm gap spacing was 20% lower than 100 μm gap spacing, and the capacitive double layer was not significantly different. The impedance data show the same trend as the CV and DCPA data for 50 μm and 100 μm gap spacing, confirming that the 50 μm gap spacing is the optimal configuration for Pt-IME under the conditions tested.

The electrochemical characterization of Pt-IME results were in disagreement with the in silico model, which did not consider cross-talk for decreased gap spacing. While decreased gap spacing theoretically enhances transport and S/N ratio, cross-talk is a major problem that has been well characterized in IDE and IME.^54^ In this study, the gap spacing of 50 μm produced the most stable response based on the portfolio analysis shown in Supplementary Table S4.

### 3.3. Pt-IME Biofunctionalization

**Figure 3:**
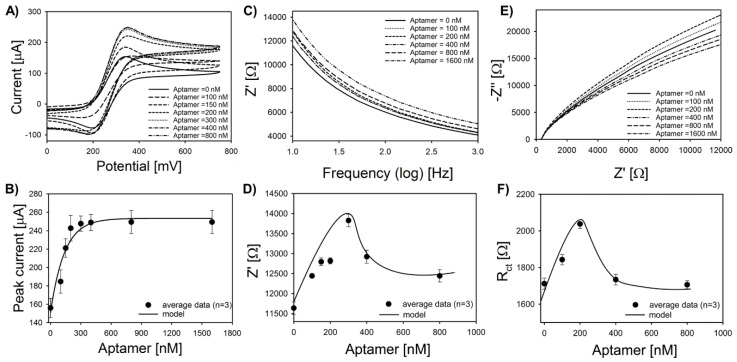
Adsorption of 47-mer on Pt-IME (50 µm gap spacing). (**A**) Representative cyclic voltammetry (CV) at different aptamer concentrations shows an increase in oxidative current after aptamer adsorption in 4 mM K_4_Fe(CN)_6_ + 1 M KNO_3_ (pH = 7.1) at room temperature. (**B**) Average peak oxidative current from CV. (**C**) Representative Bode plots at different aptamer concentrations shows an increase in impedance after aptamer adsorption. (**D**) Average net impedance at a cutoff frequency of 1 Hz from Bode plots. (**E**) Representative Nyquist plots at different aptamer concentrations shows an increase in impedance after aptamer adsorption. (**F**) Average charge transfer resistance from Nyquist plots. All error bars represent standard deviation of the arithmetic mean (n = 6 replicate electrodes). Exponential models were based on Langmuir kinetics, Frenudlich kinetics, or log normal modeling, a 4-parameter empirical model was developed for analyzing data from Bode and Nyquist plots.

Thiolated 47-mer adsorption was optimized on Pt-IME with 50 μm gap spacing. Figure 3A shows representative CVs for the adsorption study. The peak current increased when the 47-mer is adsorbed to the surface and saturated above concentrations of 600 nM. Previous studies using the same thiolated 47-mer with a 6-carbon spacer also showed that the material is conductive when adsorbed on nanoplatinum-modified electrodes [28], and this conductivity may be associated with interactions between the electrolyte and the stem loop structures [56]. Electrochemical analyses of DNA hybridization sensors have also shown that ssDNA tethered to a metal electrode can increase the electroactive surface area [57], but the magnitude depends on the total number of base pairs and location(s) of loop motifs. As shown in Figure 3B, the peak oxidative current (*i_p_*) follows pseudo-first order kinetics (Langmuir) with an adsorption capacity of 381 ± 39 nM. To analyze this behavior in more detail, impedance and charge transfer parameters were also analyzed via EIS with a sweeping frequency of 1 Hz to 100 kHz and potential bias of 200 mV.

Net impedance (Bode plots in Figure 3C) increased linearly after adsorption of the 47-mer until a concentration of 400 nM, which appears to be a critical transition point for these Pt-IME with 50 μm gap spacing. The increase in net impedance from 0 to 300 nM followed a linear regression model (R^2^ = 0.97) (Freundlich) (Figure 3D). At a concentration of 400 nM, the average net impedance decreased by 10% following a log-normal behavior, implying that a mixed adsorption model governs the kinetics. A log-normal empirical model was used to fit the data for concentrations higher than 300 nM. One potential mechanism for the mixed kinetic model transition near 400 nM is the orientation of the tethered aptamers. There is a potential for nucleobase hydrogen bonding near the upper stem loop structure (Supplementary Figure S8). Regardless of the orientation of adjacent aptamers, base pair repulsion likely occurs between the 5*′* end (location of the C6 spacer and thiol tag), which may lead to ordered deposition at low aptamer concentrations and Freundlich behavior (cooperative adsorption is based on the base pair repulsion near the attachment site). Adjacent to the larger stem loop structure at the 3*′* end, hydrogen bonding between at least two of the base pairs may cause an adsorption overshoot, as reviewed by Rabe et al. [58]. This transition from cooperative adsorption to competitive adsorption has been well described for proteins [59] and is a function of the orientation and reversible interactions away from the attachment site related nucleobase hydrogen bonding (Supplementary Figures S9 and S10). We proposed that because the bond strength of the thiol–metal (≈40 kcal mol^−1^) at the 5′ end is significantly higher than the H bonding near the upper stem loop (≈2 kcal mol^−1^), this allows the tethered aptamer to reversibly interact without loss of secondary structure. To further confirm these results, complex plane diagrams (Nyquist) were analyzed, and Nyquist plots (Figure 3C) were analyzed using equivalent circuit modeling (Randles–Ershler circuit) and also non-linear curve fitting of raw data to confirm the cooperative/competitive mixed kinetic model. Charge transfer resistance (*R_ct_*) derived from the equivalent circuit model increased linearly after 47-mer adsorption for concentrations up to 300 nM and then decreased exponentially above concentrations of 400 nM, confirming the transition from cooperative to competitive adsorption. Detailed analysis of complex plane diagrams showed that this trend was consistent for impedance values above 0.3 kΩ. Capacitive double layer (*C_dl_*) and solution resistance (*R_s_*) did not significantly change during the adsorption experiments.

The critical transition observed for the Pt-IME in this study was also shown by other studies using antibodies [60] and aptamers [61]. These results indicate that the dielectric and insulative properties of the aptamer, as well as the orientation of tethered aptamers, are important factors for electrochemical behavior. In this study, no agitation was used during biofunctionalization; thus, film diffusion may be a significant factor in the measured adsorption capacity. The adsorption experiments were repeated six times to ensure that the trends were correct, and Figure 3 shows average data with error bars representing one standard deviation of the arithmetic mean.

### 3.4. Bacteria Sensing

**Figure 4:**
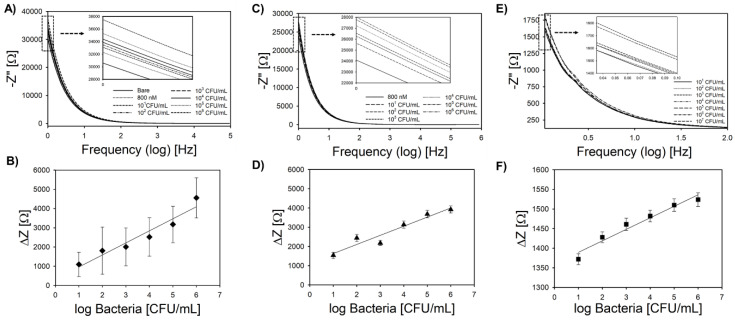
(**A**) Representative Bode plot for *L. innocua* in phosphate buffer saline (PBS) using a laboratory potentiostat under controlled conditions (inset shows linear region between 1 to 1.2 Hz). (**B**) Calibration plot using change in impedance at a cutoff frequency of 1 Hz. (**C**) Representative Bode plot for *L. monocytogenes* in in vegetable broth using a laboratory potentiostat under controlled conditions (inset shows linear region between 1 and 1.2 Hz). (**D**) Calibration plot in vegetable broth using a change in impedance at a cutoff frequency of 1 Hz. (**E**) Representative Bode plot for *L. innocua* in PBS using a smartphone-based potentiostat in hydroponic media (inset shows linear region between 10 and 100 MHz). (**F**) Calibration plot using change in impedance at a cutoff frequency of 0.03 Hz. All error bars represent standard deviation of the arithmetic mean (n = 3).

Pt-IMEs were calibrated toward *L. innocua* at room temperature using a laboratory potentiostat in PBS (pH = 7.1) and also using a handheld smartphone potentiostat in hydroponic media. Both calibrations were carried out in stagnant solution with no flow. Bode plots from the laboratory potentiostat (Figure 4A) indicate that impedance at low cutoff frequency followed a linear trend with bacteria concentration. Similar to previous work [28], the optimum cutoff frequency for bacteria capture was 1 Hz. The S/N ratio was significantly lower for frequencies higher than 2 Hz; thus, 1 Hz was used for all impedance analysis for both types of acquisition equipment. Previous studies showed that frequencies above 100 Hz correspond to the ohmic resistance of the solution [62,63], which is indicated by the convergence of the impedance curves and is also shown in this study. Normalized impedance changes versus log bacteria concentration in PBS (Figure 4B) show a linear trend (R^2^ = 0.93) with a sensitivity of 628.9 ± 148.9 Ω log-CFU^−1^ mL and an average LOD of 6 ± 1 CFU mL^−1^ for replicate sensors in buffer. The linear range was from 10^1^ to 10^6^ CFU mL^−1^. The selectivity of the biosensor toward *L. monocytogenes* was tested in a real food sample (vegetable broth). Vegetable broth was chosen as an example of complex media that presents carbohydrates and proteins, among other components, that could interact with the biosensor through non-specific adsorption resulting in a false-positive signal. Similar to the PBS measurements, the Bode plot (Figure 4C) indicated a linear relationship between impedance and bacteria concentration at low cutoff frequency. The calibration curve (Figure 4D) obtained from the impedance change versus log bacteria concentration also showed a linear range (R^2^ = 0.92) from 10^1^ to 10^6^ CFU mL^−1^ with an LOD of 7.9 ± 2 CFU mL^−1^. The sensitivity of the biosensor in vegetable broth (474.2 ± 6.1 Ω log-CFU^−1^ mL) was similar (*p* > 0.05) to the sensitivity in PBS. This result emphasizes the resilience of this biosensor even when tested in complex media.

Calibration with the handheld potentiostat developed by Jenkins et al. [29] is shown in (Figure 4E,F). The device connects to a smartphone or tablet via Bluetooth, and the data are formatted as a csv file that can be analyzed off line [29] or autonomously using support vector machine learning [34], as shown in our previous work. The sensitivity (29.3 ± 0.6 Ω log-CFU^−1^ mL) and LOD (23 ± 4 CFU mL^−1^) were significantly different than the calibration with laboratory equipment based on ANOVA analysis, which was expected given the considerable difference in circuitry and also ad hoc signal smoothing used by the commercial instrument (the handheld potentiostat reports raw signal with no signal filtering). The linear range (Figure 4F) was identical to calibration with the commercial instrument (10^1^ to 10^6^ CFU mL^−1^). The total analysis time for both tests was 17 min, including binding time, mixing, and impedimetric analysis (15 min for binding bacteria while stirring at 450 rpm).

### 3.5. Hysteresis and Reusability

**Figure 5:**
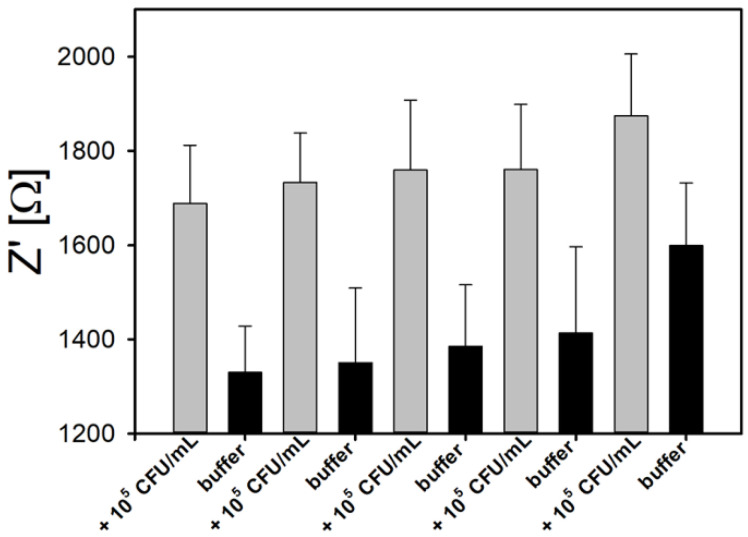
Reusability of 47-mer aptasensor after washing with NaOH at 25 °C (pH = 9.4) and rinsed in PBS (pH = 7.1). The average hysteresis after NaOH washing was 2.1 ± 2.0% for up to three reuse cycles, and 15.6 ± 6.5% after five reuse cycles. Error bars represent standard deviation of the arithmetic mean.

Aptamers have a unique capability of being reused after exposure to local conditions that induce unfolding [64]. The 47-mer used in this study is known to be stable and in the binding conformation at pH between 6.5 and 8.0, and salinity as high as 0.85% (NaCl) [21,28]. Binding with the target cell surface protein InlA is thought to be based on hydrogen bonding with the secondary/tertiary structure of the aptamer and specific groups in InlA. To test whether the aptamer was capable of reversible binding, a hysteresis experiment was conducted by recording a Bode plot in the presence of various cell concentrations, followed by a rinse in 2 N NaOH at 25 °C and a subsequent rinse in PBS for 10 min. The percent hysteresis was calculated by comparing the net impedance at 1 Hz after subsequent washing steps. The total amount of hysteresis for the Pt-IME was 15.6 ± 6.5% after five cycles and 2.1 ± 2.0% after three cycles (Figure 5). Sensors were not stable for more than five cycles, and the hysteresis was significantly higher than the range of the linear calibration curve, indicating that either cells and/or EPS (extracellular polymeric substances) were tightly bound to the surface or the aptamers were no longer adsorbed to the electrode. In addition to this simple wash cycle at relatively high pH (likely causing aptamer unfolding), other techniques such as inclusion of hairpin loop effectors [65] have been used to facilitate reversible aptamer binding in drug development studies.

A test was also conducted by washing the silicon chip with Piranha solution to remove aptamer. After 10 min of rinsing in Piranha solution, the baseline signal was 99.9% of the original values reported in Figure 2, and adsorption with aptamer was not significantly different than shown in Figure 3. The aptasensor has limited reuse due to either the aptamer detachment of hysteric folding that does not return to the native secondary structure, but the Pt-IME chip can be reused up to 20 times (at least) after washing with Piranha solution (Supplementary Table S5).

### 3.6. Analysis of Hydroponic Water in Particle Trap Filter

**Figure 6:**
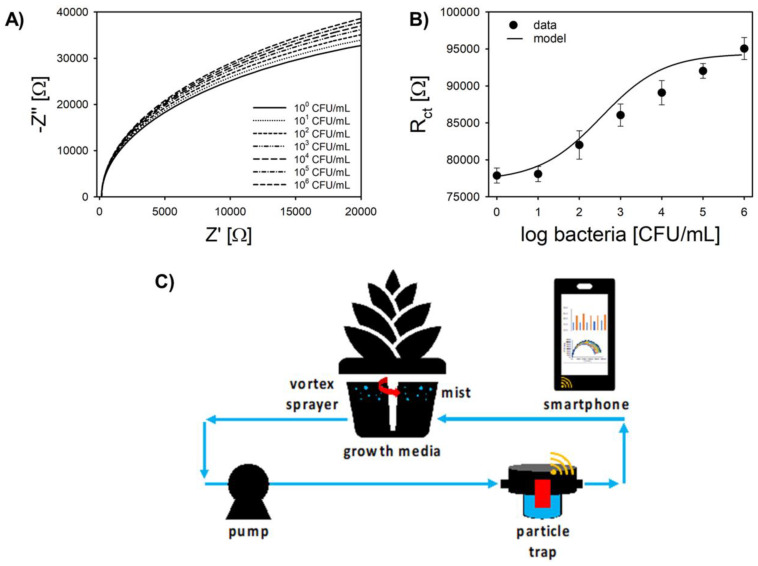
Calibration of Pt-IME biosensor in hydroponic growth media for *Listeria innocua* in the particle flow trap. (**A**) Representative Nyquist plot for increasing concentrations of *Listeria innocua* in growth media. (**B**) Calibration plot using ΔR_ct_ (Ω) and logistic regression curve. Dashed lines show the 99% confidence interval for the logistic curve. Error bars represent standard deviation of the arithmetic mean (n = 3). (**C**) Schematic of flow-through system with aptasensor (red) in particle trap connected to a smartphone potentiostat. See Supplementary Figure S11 for photograph.

Figure 6A,B shows a representative Nyquist plot and calibration curve for the Pt-IME aptasensor targeting *L. innocua* in the particle/sediment trap under flow conditions. To our knowledge, this is the first demonstration of in line flow-through analysis of hydroponic media for bacteria testing with relatively large volumes (100 mL). A sampling system pumped solution from the tank of the hydroponic system into the particle trap at a flowrate of 10 mL min^−1^, and the pump was stopped after a volume of 100 mL was processed (Figure 6C). After pumping, the Pt-IME was allowed to stabilize for 15 min, and the EIS analysis required 2 min (total response time was 27 min).

Using charge transfer resistance (R_ct_) derived from the model shown in Equation (2), the aptasensor sensitivity in the flow-through system was 3.37 ± 0.21 kΩ log-CFU^−1^ mL, and the LOD was 48 ± 12 CFU mL^−1^ (Figure 6B). The linear range was 10^2^ to 10^4^ CFU mL^−1^. All of the performance characteristics were significantly different than the analysis in PBS buffer or in hydroponic solutions with no flow. Most importantly, there was a clear zero order region from 0 to 10 CFU mL^−1^, which was characterized by the logistic function shown in Figure 6B. If a linear calibration curve is used according to common convention, the data below 2-log CFU mL^−1^ cannot be quantified (LOD using a S/N ratio of 3 was 48 CFU mL^−1^ and the limit of quantitation was 100 CFU mL^−1^). On the other hand, a logistic function was developed as the calibration curve and confidence intervals were used to quantify concentrations as low as 1 CFU mL^−1^. Recent advancements in data science have demonstrated the ability to use advanced techniques on smartphones, including machine learning approaches based on impedance data from biosensors [34]. Consequently, it is highly conceivable that the non-linear approach shown in Figure 6 can be further developed to analyze sensor data in flow-through systems and achieve lower limits of detection in real samples using volumes of 100 mL or greater. For such an approach, additional time for data analysis is required for the model to process the raw impedance data and calculate the R_ct_ by fitting with the parameters derived from calibration.

Table 1 shows the measured values and recovery (%) for data collected after spiking the hydroponic system with *L. innocua* concentrations of approximately 200 CFU mL^−1^ (above the limit of detection and the limit of quantitation). In repeated trials, the average recovery was 90 ± 4%, and all spiked samples were significantly different than control samples (*p* < 0.05). The flow-through system is capable of monitoring *Listeria* spp. in hydroponic media using the grab sample approach here, and it can be improved by developing simple management tools such as an artificial reasoning tool (ART) for rapid decision support [44,66].

### 3.7. Comparison of Listeria Sensors in the Literature

A wide range of electrochemical, optical, and mass-based nanobiosensor devices have been reported in the literature for the detection of *Listeria* spp., all with varying levels of performance. Sensors based on optical transduction (fluorescence, colorimetry, surface plasmon resonance) [20,21] or piezoelectric transduction [67] have been tested in various food samples. While the LOD of fluorescent and colorimetric sensors has improved in the last few decades, the response time tends to be significantly slower than electrochemical sensors. For example, the aptamer sandwich assay developed by Lee et al. [68] presented an LOD of 20 CFU mL^−1^ but a response time of 2 h in buffer. The lowest LOD for colorimetric sensors was 2.4 CFU mL^−1^, but it required 6.5 h in buffer [19]. Few SPR-based devices for *Listeria* detection are found in the literature, but Boulade et al. [69] recently reported a SPR-based device with a LOD of 2 × 10^2^ CFU mL^−1^ after 7 h.

The biorecognition materials for targeting extracellular target(s) on *Listeria* spp. include antibodies [46,70,71,72], aptamers [21,68], and peptides [73]. Other approaches utilized endolysin [46] or soluble proteins for the indirect monitoring of metabolic biomarkers [74]. Endolysin devices require pre-conditioning prior to measurement (can take up to 16 h) and have issues with selectivity. The measurement of biomarker metabolites is valuable for detecting cell growth in specific media (e.g., Tris-Gly-Dext). However, the devices such as the design by Gómez et al. [74] have limited selectivity toward *Listeria* spp., the analysis time is long, and the range is narrow at high bacteria concentrations (1.9 × 10^7^ to 3.8 × 10^7^ CFU mL^−1^). Among the material used as recognition structure in biosensors for *Listeria*, aptamers have well-documented advantages, including longer shelf life, enhanced durability, and lower cost [75,76,77].

Among electrochemical biosensors, impedimetric devices, such as the Pt-IME developed here, are the most common (a select number of devices is shown in Table 2). Wang et al. [70] demonstrated a device with a linear range (10^2^ to 10^7^ CFU mL^−1^) similar to the Pt-IME in this study; however, the analysis time and LOD were significantly higher than other devices in the literature. This is a major problem, as the analysis time is critical to rapid diagnostics under the USDA “zero tolerance” and “hold and test” policy on food contact surfaces and ready-to-eat products [78]. The lowest LOD for impedimetric biosensors in food samples without using a microfluidic device was 5 CFU mL^−1^ in tomato extract [79]. This device was based on the immobilization of antibodies on gold-coated glass slides. Hills et al. [28] also reported an LOD below 10 CFU mL^−1^ using stimulus-responsive nanobrushes and a DNA aptamer in vegetable broth. Although these devices did not use microfluidics, both sensors depend on direct exposure to spiked pathogen samples in small volumes (under 20 mL).

The Pt-IME device reported here has similar performance in controlled conditions. When comparing impedimetric devices in PBS buffer, the Pt-IME aptasensor had a lower detection limit (6 ± 1 CFU mL^−1^), higher sensitivity (628.9 ± 148.9 Ω log-CFU^−1^ mL), shorter analysis time (17 min), and wider operating range (10^1^ to 10^6^ CFU mL^−1^) compared to other similar biosensors in the literature. Moreover, this biosensor presented similar sensitivity (474.2 ± 6.1 Ω log-CFU^−1^ mL) toward *L. monocytogenes* in complex media (vegetable broth), with an LOD of 7.9 ± 2 CFU mL^−1^ and sensing range from 10^1^ to 10^6^ CFU mL^−1^. When washed with Piranha solution, the sensor had 0% hysteresis, and when treated with NaOH and washed with DI water, the hysteresis ranged from 2 to 16%. The characterization of signal hysteresis is the foundation for determining sensor reusability, which is critical for the development of future tools such as the artificial reasoning tool (ART) [44,66]. Furthermore, the sensor reported here is able to monitor *Listeria* spp. in a high flow-through system for on-site analysis of water quality in hydroponics. The resulting system showed a response time of 27 min at relevant concentration ranges (10^2^ to 10^4^ CFU mL^−1^). Limitations of this sensor system are related to the significant increase in the LOD from 6 ± 1 CFU mL^−1^ to 48 ± 12 CFU mL^−1^ for stagnant and high flow-through media, respectively, and the limited number of direct reuses (i.e., treating with NaOH solution and rising with DI water).

There are many devices targeting *Listeria* spp. in microfluidic systems [22,71,74,80,81,82], and these laboratory assays are excellent for validation purposes after rapid screening. For example, Etayash et al. [73,83] demonstrated the detection of one cell in volumes as small as 0.01 mL. However, in the context of real-time decision support, these devices are currently expensive, require user expertise training, and contain multiple disposable components. For example, Chiriacò et al. [82] recently reported an LOD of 5.5 CFU mL^−1^ with a microfluidic impedimetric system on a gold-IME immunosensor, utilizing a portable potentiostat. However, this device has low throughput (20 µL sample per hour).

The Pt-IME developed here is the first demonstration of a sensor for direct, label-free analysis in a flow-through system at high flow-through volume (100 mL). The most practical path forward may be to blend various sensors to meet industry needs using a distributed platform system such as the framework for sensor-analytic point solutions by McLamore et al. [44]. While sensor data alone do not provide a preventative measure, they do provide crucial information; when combined with good agricultural practices, hygienic practices, and storage practices, this tool is a vital step forward to controlling *Listeria* contamination in fresh lettuce.

## 4. Conclusions

The rapid, label-free aptasensor developed here represents an important step forward in the development of tools for assessing agricultural water quality. The real-time (27 min) *Listeria* sensor was applied to hydroponic media for sample volumes up to 100 mL. The use of a commercially available handheld, smartphone-based acquisition system and off-the-shelf components to develop the particle trap/sensor system ensure that the sensor system can be recreated by other sensor labs around the world. Potential management strategies may involve a rapid, high volume flow-through sensor for screening such as the Pt-IME shown here, followed by the secondary validation of high-risk samples to ensure water meets regulatory standards. A “portfolio approach” using multiple sensors is often necessary for simultaneously meeting economic and monitoring needs of the food/human health industries and regulatory agencies to ensure food safety and public health.

Future work includes expanding the list of relevant microorganisms to hydroponic leafy vegetables production that can be monitored using the sensing platform shown here. These include generic *Escherichia coli* as an indicator organism and pathogenic bacteria such as *Salmonella enterica* serovars, and Shiga toxin-producing *E. coli* (STEC), which are among the most common cause of gastroenteritis associated with fresh produce [86]. Each of these pathogens has characteristics that enable their survival in the built environment of hydroponic systems for extended time periods [87,88]. Furthermore, validation of the sensing platform shown here using large-scale hydroponic systems would be beneficial to translate to a real-life scenario. Additionally, strategies to further improve the limit of detection reported herein should be explored. One strategy is to enhance bacteria capture using polymer brushes that actuate under environmental stimuli, as demonstrated previously by Hills et al. [28]. In order to implement this approach in hydroponic systems, the development of a cyber–physical system is required to allow actuation at multiple scales (macro/nano).

## Figures and Tables

**Table 1:** Summary of Pt-IME flow-through analysis of hydroponic lettuce system. *Listeria innocua* was spiked into the system at known concentrations and pumped through a particle trap with an embedded Pt-IME biosensor. After 5 min of continuous flow, the pump was turned off for electrochemical impedance spectroscopy (EIS) analysis with a handheld potentiostat. Data were analyzed using an equivalent circuit model for calculating R_ct_.

Added [CFU mL^−1^] ^a^	R_ct_; Spiked Sample [kΩ] ^b^	R_ct_; Control [kΩ] ^b^	Measured [CFU mL^−1^]	Rec. ^c^ [%]
219	83.1	78.0	210	96
230	83.1	78.1	215	94
232	83.0	78.4	198	86
351	83.9	77.9	390	89
217	83.0	77.9	195	90
221	83.3	78.2	246	89

^a^ Spiked concentration determined by OD600; ^b^ Charge transfer resistance calculated with model shown in Equation (2); ^c^ Rec. = Recovery.

**Table 2:** Comparison of impedimetric biosensors for the detection of *Listeria* spp.

Microelectrode (Rec. Element ^a^)	Sample	Time [min]	LOD [CFU mL^−1^]	Linear Range [CFU mL^−1^]	Hysteresis [%]	Ref.
TiO_2_ nanowire bundle (Ab)	buffer	50	470	10^2^ to 10^7^	NR	Wang et al. [70]
Screen printed electrode (Ab-NPs ^b^ + AuNPs ^c^)	blueberry	60	231	10^3^ to 10^6^	NR	Davis et al. [84]
Au-IME with portable potentiostat (Ab)	milk	60	5	10^2^ to 10^3^	NR	Chiriacò et al. [82]
Gold screen printed electrode (endolysin)	milk	30	1.1 × 10^5^	10^5^ to 10^9^	NR	Tolba et al. [46]
Screen printed IDE	lettuce	180	1.0 × 10^3^	10^3^ to 10^6^	NR	Wang et al. [85]
nPt ^d^-rGO ^e^ electrode (InlA aptamer)	vegetable broth	17	9.1	10^1^ to 10^7^	NR	Hills et al. [28]
Pt-IME with laboratory potentiostat (InlA aptamer)	buffer	17	6 ± 1	10^1^ to 10^6^	15.6%	This study
laboratory potentiostat (In1A aptamer)	vegetable broth	17	7.9 ± 2	10^1^ to 10^6^	NR	This study
Pt-IME with smartphone potentiostat (InlA aptamer)	hydroponic media	27	23 ± 4	10^2^ to 10^6^	15.6%	This study
Flow through Pt-IME with smartphone potentiostat (InlA aptamer)	hydroponic media	27	48 ± 12	10^2^ to 10^4^	24.9%	This study

^a^ Rec. element = biorecognition element; ^b^ Ab-NPs = immunomagnetic nanoparticles; ^c^ AuNPs-urease = gold nanoparticles functionalized with urease; ^d^ nPt = nanoplatinum; ^e^ rGO = reduced graphene oxide; NR = not reported.

## Supplementary Materials

The following are available online at https://www.mdpi.com/1424-8220/20/20/5773/s1, Figure S1: general design schematic for platinum interdigitated microelectrodes (Pt-IME), Figure S2: IME incorporated into particle flow trap for continuous analysis, Figure S3: the gap size of 50 μm electrode array with Dektak profilometer measurement, Figure S4: (A) IME model output for various gap spacing (COMSOL). (B) comparison of measured and predicted capacitance for various gap spacing in buffer, Figure S5: (A) estimation of cell constant and electrode spacing for IME (B) Olthius plot (C) simulation of electrical field at the surface of IME, Figure S6: representative plots of electrochemical characterization of Ti/Pt IME with different gap spacing, Figure S7: representative DCPA for Pt-IME with 50 (A, B) and 100 (C, D) μm gap spacing, Figure S8: cartoon representation of secondary structure predicted using mfold, Figure S9: cartoon representation of ferricyanide redox probe near the surface of Pt-IME with no aptamers, Figure S10: cartoon representation of redox probe used to measure electrochemical behavior, and Figure S11: (A) photograph of hydroponic system with Pt-IME. (B) Pt-IME incorporated into particle flow trap for continuous analysis. Table S1: materials and design dimensions for Pt-IME fabrication, Table S2: design characteristics of IME with various spacing and measured physical features using Dektak profilometer, Table S3: summary of electrochemical characterization using ferrocyanide as the redox probe, Table S4: portfolio analysis for IME with various gap spacing, and Table S5: cleaning electrodes with Piranha solution.
