# Supplementary material for: Planar Interdigitated Aptasensor for Flow-Through Detection of Listeria spp. in Hydroponic Lettuce Growth Media
Source: Sensors (Basel). 2020 Oct 12;20(20):5773. doi: 10.3390/s20205773 (PMC7600482; doi:10.3390/s20205773)
Supplement: Supplementary file 1 [file sensors-20-05773-s001.pdf]

Supplemental Section

Planar interdigitated aptasensor for flow-through detection of Listeria spp. In hydroponic lettuce growth media

Raminderdeep K. Sidhu[a], Nicholas D. Cavallaro[b], Cicero C. Pola[c], Michelle D. Danyluk[d], Eric S. McLamore[b*], Carmen L. Gomes[c*],

[a]Department of Biological & Agricultural Engineering, Texas A&M University, College Station, TX 77843, USA

[b]Agricultural & Biological Engineering, Institute of Food and Agricultural Sciences, University of Florida, Gainesville, FL 32611, USA

[c]Department of Mechanical Engineering, Iowa State University, Ames, IA 50011, USA

[d]Food Science and Human Nutrition, Institute of Food and Agricultural Sciences, University of Florida, Gainesville, FL 32611, USA

[*]corresponding authors: carmen@iastate.edu; emclamore@ufl.edu

Table S1. Materials and design dimensions for Pt-IME fabrication.

Design Element	Dimensions
Substrate	4 inch SiO_2 with 300 nm thermal oxide layer
Active area	0.81 cm^2
Electrode width	25 μm
Electrode gap	Variable: 15, 25, 50, 100 μm as noted
Bonding pads	2mm X 2mm
Ti layer thickness	15 nm
Pt layer thickness	100 nm

Table S2. Design characteristics of IME with various spacing and measured physical features using Dektak profilometer.

Design Feature	Design Specifications	Actual Dimensions	Percent difference
gap	25 μm	12 to 15	40 to 52
gap	50 μm	35 to 40	20 to 30
gap	100 μm	82 to 87	13 to 18
width	25 μm	22 to 27	8 to 12
Ti/Pt metal layer thickness	110 nm	110 to 115	0 to 5

Table S3. Summary of electrochemical characterization using ferrocyanide as the redox probe. Values are shown as mean ± standard deviation (n= 3 independent replicates).

Electrode gap [μm]	Electroactive surface area (ESA) [cm^2]	Sensitivity toward H_2O_2 [μA mM^{-1}]	HET Constant [cm s^{-1} X10^{-4}]	Current Density [μA mM^{-1} cm^{-2}]
25	$0.04_a \pm 0.01$	COv	$7.9_a \pm 6.4$	COv
50	$0.14_b \pm 0.02$	$21.3_a \pm 0.1$	$34.6_b \pm 9.1$	$149_a \pm 20$
100	$0.11_c \pm 0.02$	$5.4_b \pm 0.1$	$44.2_b \pm 10.2$	$75_b \pm 2$

$_{a,b,c}$ means within a column which are not followed by a common subscript are significantly different ($p<0.05$).

COv = data not stable due to charge overflow

Average ESA was calculated using the mean of oxidation and reduction peaks for three replicate Pt-IMEs.

Table S4. Portfolio analysis for IME with various gap spacing. Weighting factors are normalized for a maximum score of 100.

gap spacing	S^*C_S	C^*C_C	H^*C_H	ESA^*C_{ESA}	I^*C_I	Z^*C_Z	Z^*C_{Ef}	Cumulative score
25	2.6	14.9	1.6	4.0	7.1	4.0	9.9	44.0
50	2.6	6.0	6.9	14.0	20.6	2.4	8.8	61.2
100	0.7	3.0	8.8	11.0	4.4	1.1	7.0	36.0

C_S = weighting factor for sensitivity toward H_2O_2 [μA^{-1} mM] (0.125);
C_C = weighting factor for current density [μA^{-1} mM^{-1} cm^2] (0.040);
C_H = weighting factor for HET constant [cm^{-1} s^1 $X10^4$] (0.200);
C_{ESA} = weighting factor for electroactive surface area [cm^{-2}] (0.30);
C_I = weighting factor for peak current [μA^{-1}] (0.029);
C_Z = weighting factor for impedance at 1 Hz [Ω^{-1}] (0.025); and
C_{Ef} = weighting factor for electric field [m V^{-1}] (0.003);

Table S5. Cleaning electrodes with Piranha solution. Protocol for cleaning and maximum current shown.

Piranha cleaning time	electrode configuration	scan rate [mV/s]	Maximum current [µA]	solution color
Virgin	3 electrode & sensing not connected	50	183.3	white
10 min	4 electrode w/0 checking the 4-electrode box	50	186.3	white
10 min	4 electrode w/0 checking the 4-electrode box	50	187.3	yellow
10 min	3 electrode w/ dbl layer ON	100	225.5	yellow
30 min	3 electrode w/ dbl layer ON	50	176.5	white
30 min	4 electrode w/0 checking the 4-electrode box	50	182.1	yellow
30 min	3 electrode w/ dbl layer ON	100	222.8	yellow

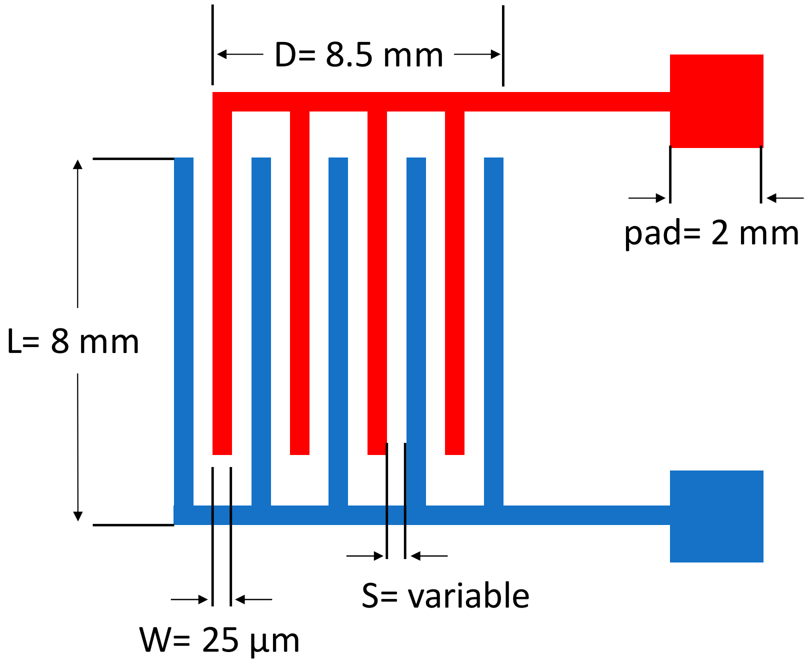

Figure S1. General design schematic for platinum interdigitated microelectrodes (Pt-IME). Gap spacing (S) design values of 25, 50, and 100 µm were used in this study.

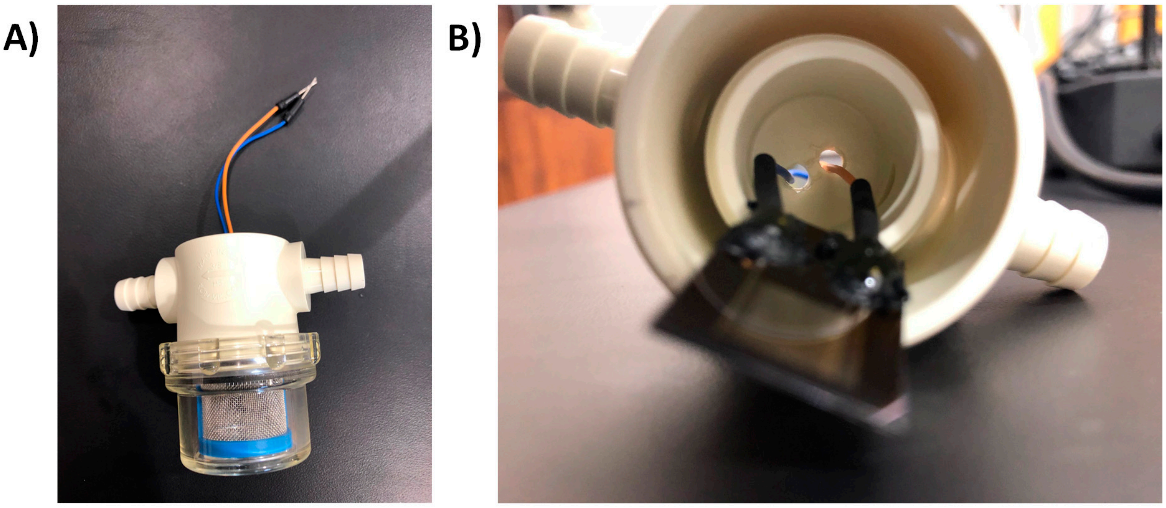

Figure S2. IME incorporated into particle flow trap for continuous analysis. **A)** Photograph of nylon particle trap with stainless steel filter screen (304 micron, grade 50 mesh). The dimensions of the trap are 3" L x 1.89" W x 2.67" H, and **B)** Photograph of Pt-IME in the particle trap.

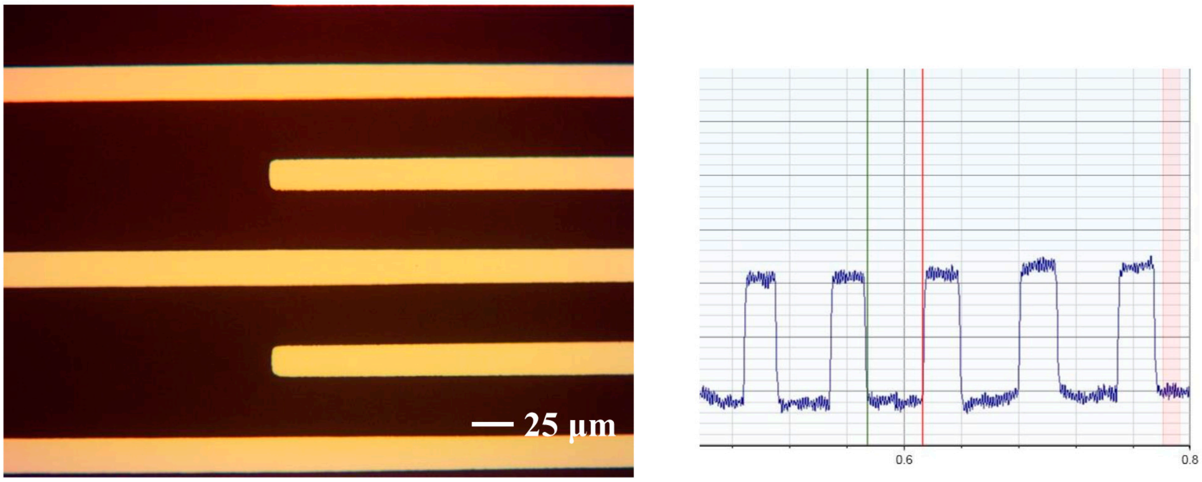

Figure S3. The gap size of 50 µm electrode array with Dektak profilometer measurement.

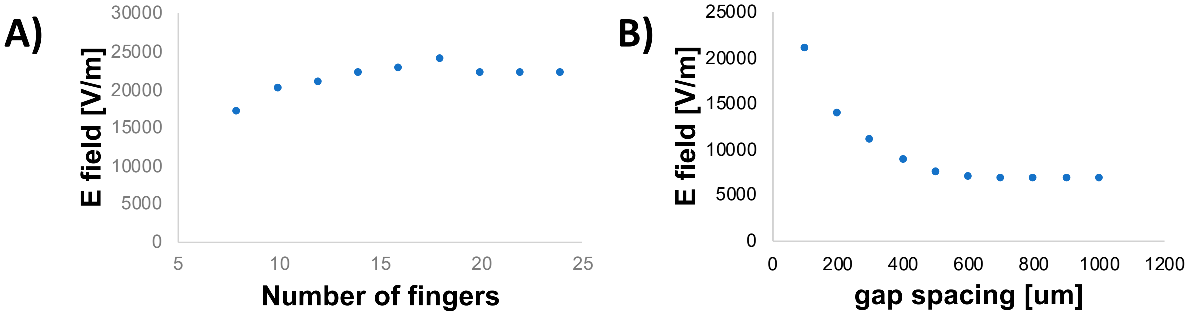

Figure S4. A) IME model output for various gap spacing (COMSOL). **B)** Comparison of measured and predicted capacitance for various gap spacing in buffer.

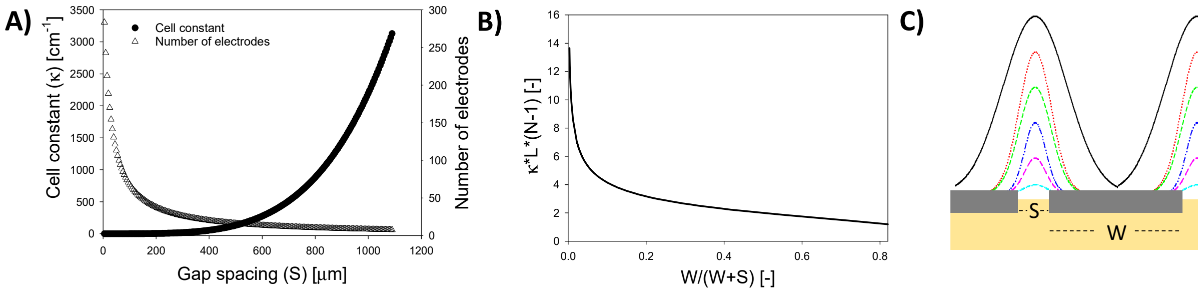

Figure S5. **A)** Estimation of cell constant and electrode spacing for IME **B)** Olthius plot **C)** Simulation of electrical field at the surface of IME.

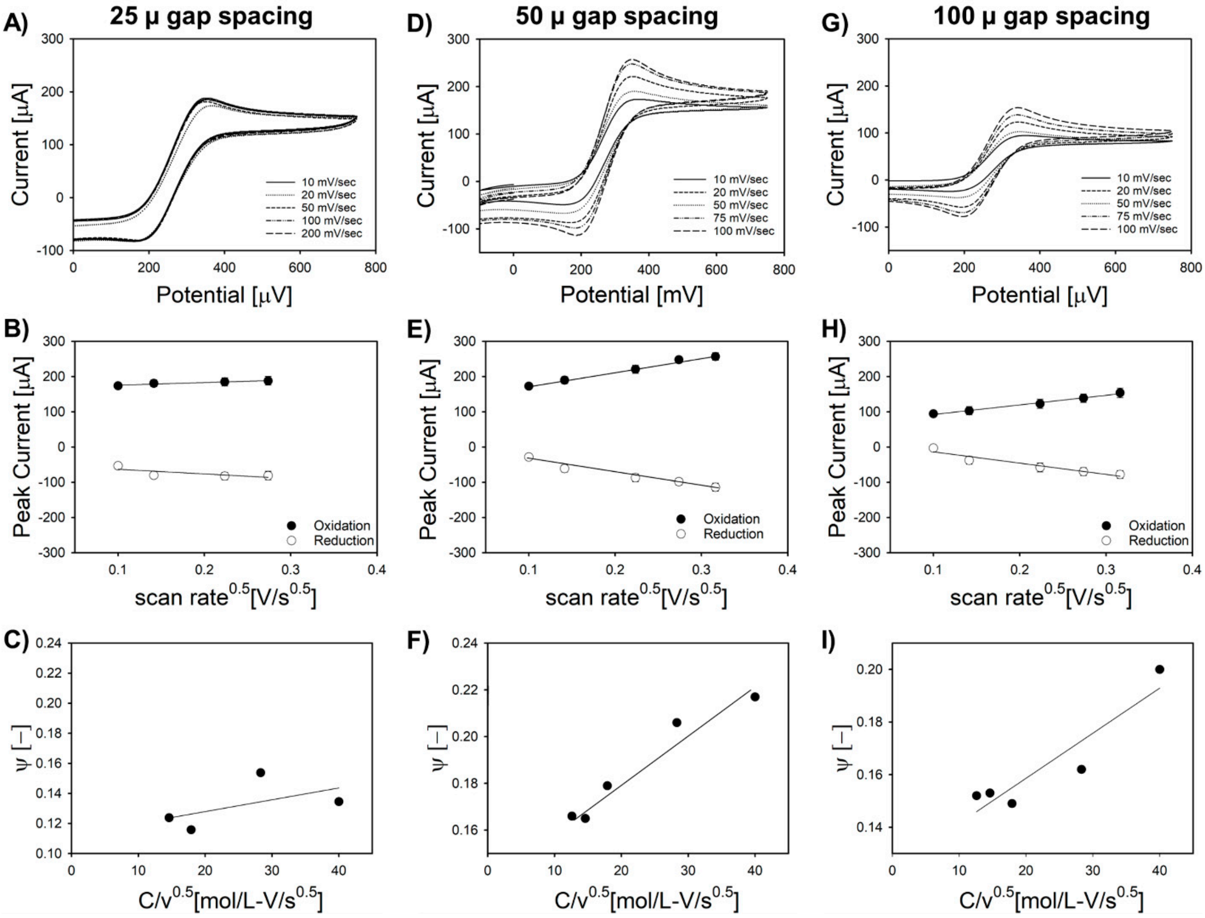

Figure S6. Representative plots of electrochemical characterization fo Ti/Pt IME with different gap spacing. Panels are organized as follows: (**A-C**) gap spacing of 25 µm, (**D-F**) gap spacing of 100 µm, (**G-I**) gap spacing of 100 µm. Top row-Cyclic voltammograms in 4mM K_3FeCN_6 at room temperature (pH=7.1). Middle row- Randles-Sevcik plots for oxidative and reductive peak current. Bottom row-Nicholson plots for determination of k^0. Average data for all IMEs is shown in Table S3.

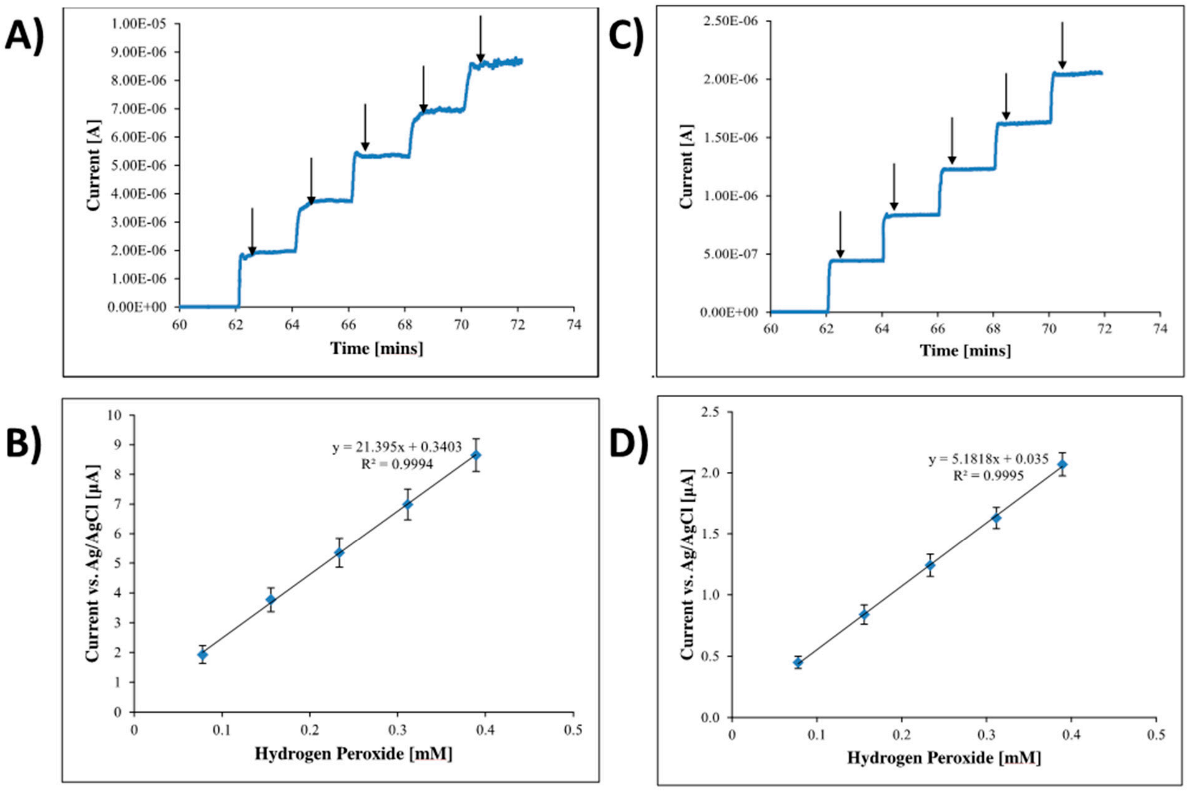

Figure S7. Representative DCPA for Pt-IME with 50 (**A, B**) and 100 (**C, D**) µm gap spacing. Average data for all IMEs is shown in Table S3.

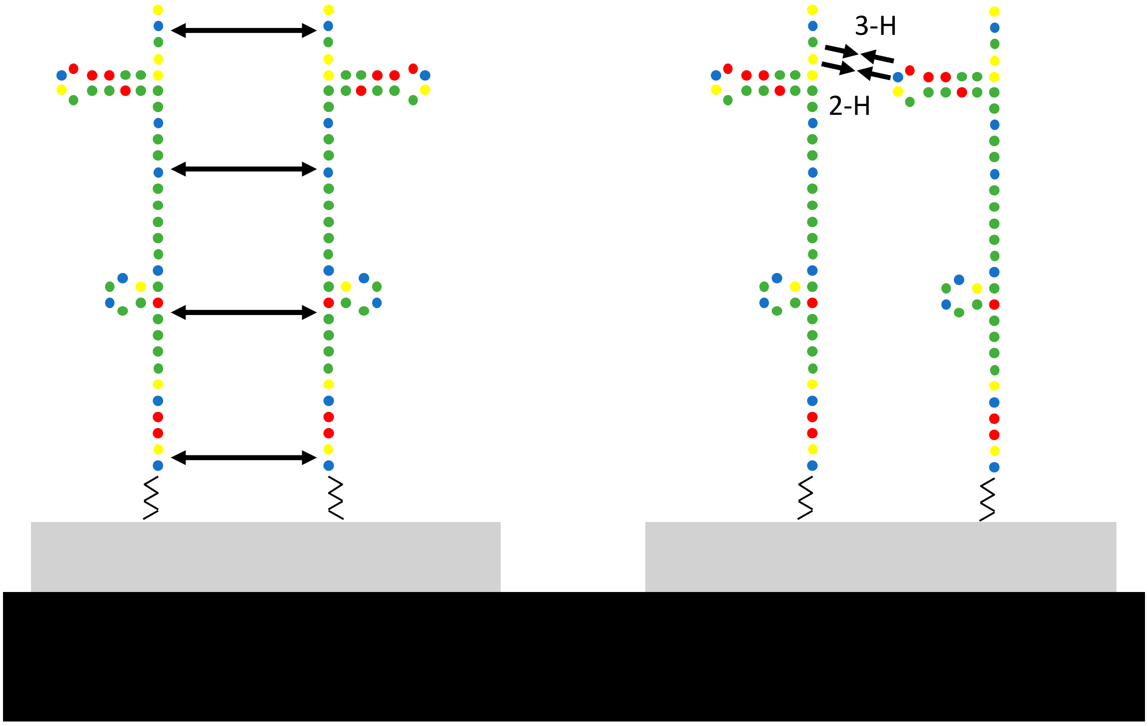

Figure S8: Cartoon representation of secondary structure predicted using mfold. (left) Potential repulsion between base pairs when multiple aptamers are in mirror conformation. (right) Potential hydrogen bonding between the upper stem loop structure when multiple aptamers are in ordered conformation. The bond strength of the thiol-metal (≈40 kcal/mol)at the base tether is significantly higher than the H bonds (≈2 kcal/mol) near the upper stem loop, indicating that any H bonding in the ordered conformation is likely reversible. This dynamic interaction likely plays a role in the measured electrochemical behavior.

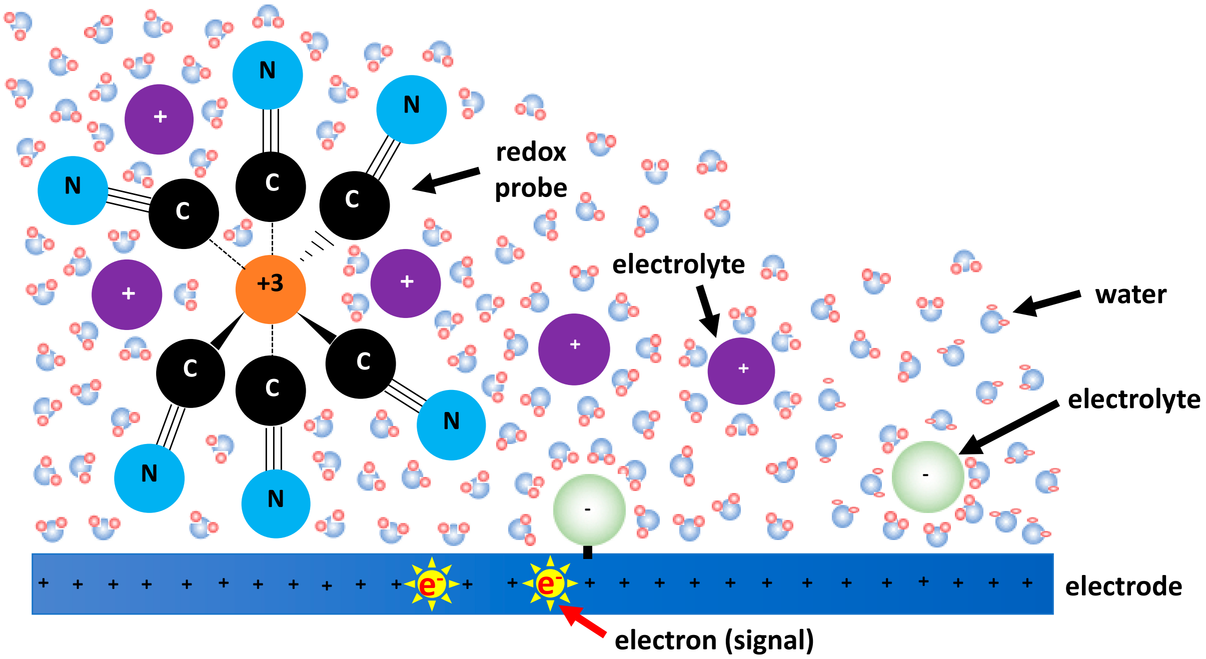

Figure S9: Cartoon representation of ferricyanide redox probe near the surface of Pt-IME with no aptamers. The redox probe orients near the electrode surface and undergoes oxidation to ferrocyanide via a single potassium ion (a one electron exchange reaction). Water hydrates electrolyte and redox probe within the dielectric layer.

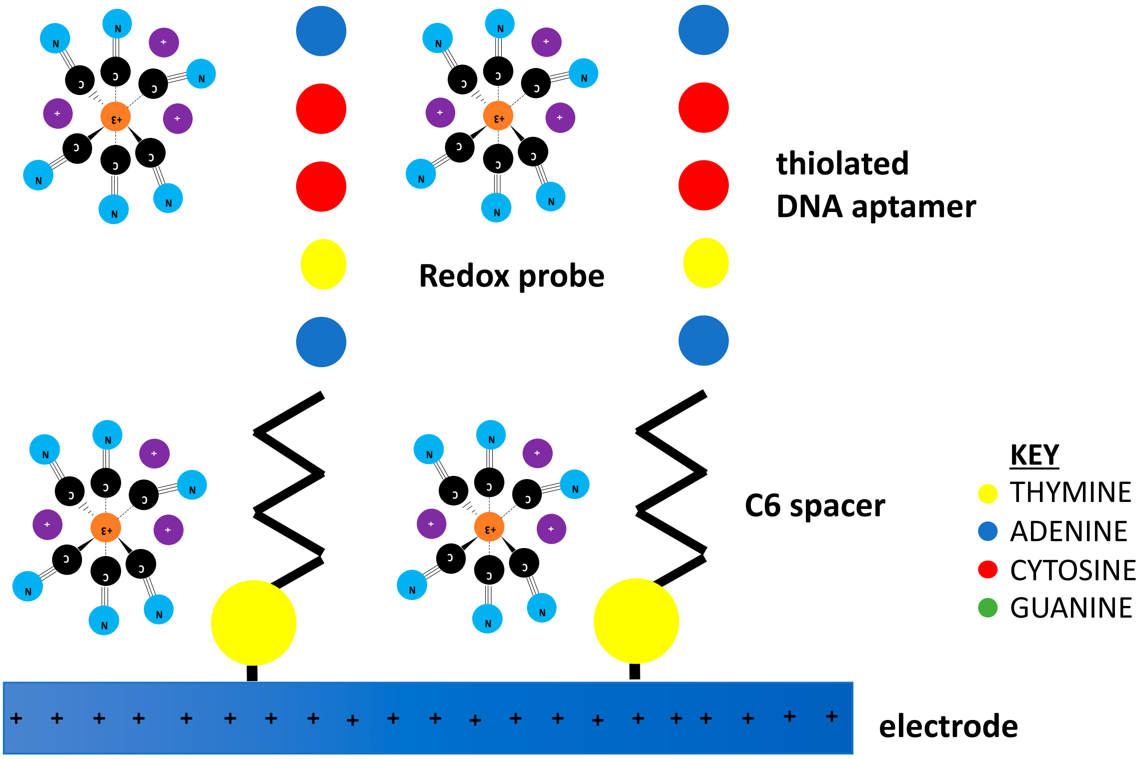

Figure S10. Cartoon representation of redox probe used to measure electrochemical behavior. Nucleobase are represented by spheres according to the color legend (drawing not to scale). Near the surface, no nucleotide interactions occur due to base pair repulsion and the 2D tethering of aptamers is assumed to be locally ordered as depicted in the cartoon.

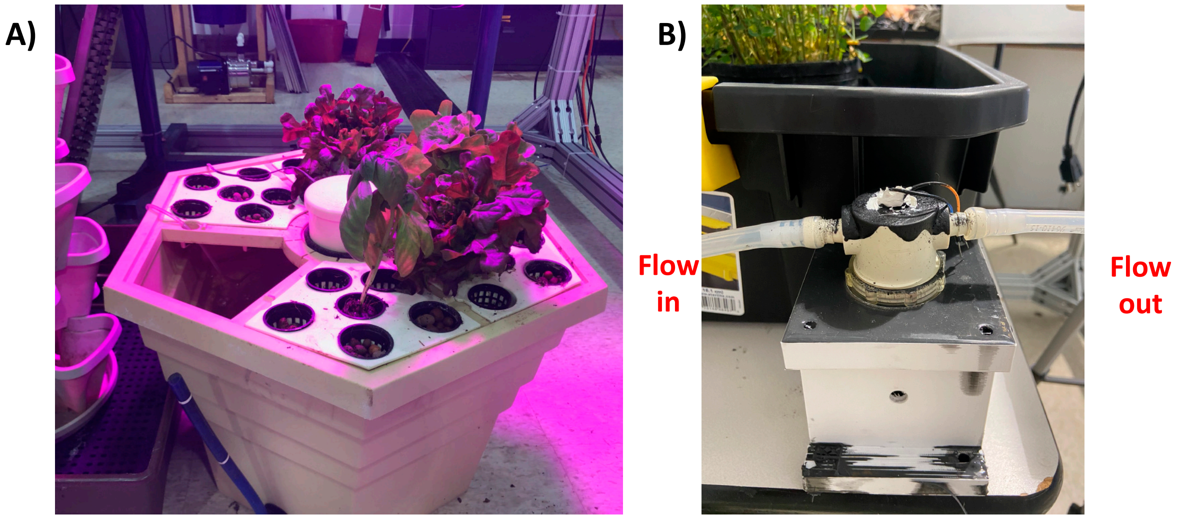

Figure S11. **A)** Photograph of hydroponic system with Pt-IME. **B)** Pt-IME incorporated into particle flow trap for continuous analysis.

© 2020 by the authors. Licensee MDPI, Basel, Switzerland. This article is an open access article distributed under the terms and conditions of the Creative Commons Attribution (CC BY) license (http://creativecommons.org/licenses/by/4.0/).
